# Adipose segmentation in small animals at 7T: a preliminary study

**DOI:** 10.1186/1471-2164-11-S3-S9

**Published:** 2010-12-01

**Authors:** Yang Tang, Susan Lee, Marvin D Nelson, Simerly Richard, Rex A Moats

**Affiliations:** 1 Department of Radiology, University of Southern California, Childrens Hospital Los Angeles, Los Angeles, USA; 2 Department of biomedical engineering, University of Southern California, Childrens Hospital Los Angeles, Los Angeles, USA; 3 Department of Paediatrics and Biology, University of Southern California, Childrens Hospital Los Angeles, Los Angeles, USA

## Abstract

**Background:**

Small animal MRI at 7 Tesla (T) provides a useful tool for adiposity research. For adiposity researchers, separation of fat from surrounding tissues and its subsequent quantitative or semi- quantitative analysis is a key task. This is a relatively new field and a priori it cannot be known which specific biological questions related to fat deposition will be relevant in a specific study. Thus it is impossible to predict what accuracy and what spatial resolution will be required in all cases and even difficult what accuracy and resolution will be useful in most cases. However the pragmatic time constraints and the practical resolution ranges are known for small animal imaging at 7T. Thus we have used known practical constraints to develop a method for fat volume analysis based on an optimized image acquisition and image post processing pair.

**Methods:**

We designed a fat segmentation method based on optimizing a variety of factors relevant to small animal imaging at 7T. In contrast to most previously described MRI methods based on signal intensity of T1 weighted image alone, we chose to use parametric images based on Multi-spin multi-echo (MSME) Bruker pulse sequence which has proven to be particularly robust in our laboratory over the last several years. The sequence was optimized on a T1 basis to emphasize the signal. T2 relaxation times can be calculated from the multi echo data and we have done so on a pixel by pixel basis for the initial step in the post processing methodology. The post processing consists of parallel paths. On one hand, the weighted image is precisely divided into different regions with optimized smoothing and segmentation methods; and on the other hand, a confidence image is deduced from the parametric image according to the distribution of relaxation time relationship of typical adipose. With the assistance of the confidence image, a useful software feature was implemented to which enhances the data and in the end results in a more reliable and flexible method for adipose evaluation.

**Results:**

In this paper, we describe how we arrived at our recommended procedures and key aspects of the post-processing steps. The feasibility of the proposed method is tested on both simulated and real data in this preliminary research. A research tool was created to help researchers segment out fat even when the anatomical information is of low quality making it difficult to distinguish between fat and non-fat. In addition, tool is designed to allow the operator to make adjustments to many of the key steps for comparison purposes and to quantitatively assess the difference these changes make. Ultimately our flexible software lets the researcher define key aspects of the fat segmentation and quantification.

**Conclusions:**

Combining the full T2 parametric information with the optimized first echo image information, the research tool enhances the reliability of the results while providing more flexible operations than previous methods. The innovation in the method is to pair an optimized and very specific image acquisition technique to a flexible but tuned image post processing method. The separation of the fat is aided by the confidence distribution of regions produced on a scale relevant to and dictated by practical aspects of MRI at 7T.

## Background

Increased adiposity is a risk factor for many diseases. Unfortunately obesity has become a significant risk factor not only in the United States, but also for the entire globe due to increase caloric intake in many regions. Magnetic resonance imaging (MRI) allows researchers to study the physiological and pharmacological effect of obesity in small animal models. Fat volume quantification plays an important role in the research of obesity induced diseases; this paper will focus on a reliable fat separation method utilizing a typical NMR sequence as opposed to a more exotic chemical shift based methods not yet implemented at high field strengths in small animal imaging in this case specifically at 7T. In addition routine sequences offer higher resolution than chemical shift based methods with resolution being an important factor in small animals.

To date various MRI segmentation methods for fat quantification have been reported [1-4]. However, most of these methods depend only on signal intensity in the weighted images. These methods are based on the assumption that fat tissues in MRI correspond to the high intensity, which introduces uncertainty as fat signals do not actually always represent the highest portion of the intensity histogram (e.g. a tissue with higher proton density). Intensity of MRI is a complex function of many factors including the magnetic field strength, image acquisition technique and acquisition parameters. Therefore, the same region of fat in two different MRI scan protocols may not have the same intensity. Finally, magnetic field inhomogeneity may subtly distort most weighted images especially at higher field strengths, resulting in different intensities for the same tissues within the same scan.

In contrast to a weighted image, the parametric image (transverse relaxation time of each pixel) is calculated from the multi-echo images. The decay of the intensity for each pixel is dependent on the physical feature (relaxation time) and should be independent of the acquisition technique at a given field strength. The calculated relaxation times represent biological characteristics of specific tissue, and can serve as the basis of fat separation [5,6]. Parametric images provide more reliable approximations of pixels contain fat tissue.

Parametric images, however, are easily corrupted by noise and artifacts derived from instrumentation as well as subject animals themselves. In addition, partial volume [7] occurs in many images, blurring the boundary of different tissues, which interfere with the precise segmentation directly from a parametric map. Dixon methods [8] address the fat suppression problem based on chemical shift but have never been implemented at 7T in a small animal scanner to our knowledge. Thus these techniques are beyond the scope of this paper.

Our approach described in this paper is based on the combination of the T1 and T2 optimized first echo images(called first image later) and the parametric images based on the multiply echo acquisition. On one hand, the first image is precisely divided into different regions with optimized smoothing and segmentation methods; and on the other hand, a confidence image is calculated from the parametric image according to the distribution of relaxation time relationship of typical fat from phantoms and test animals. With the addition of the confidence image, a useful software tool was created to offer a reliable and flexible method for fat segmentation.

## Methods

**Figure 1:**
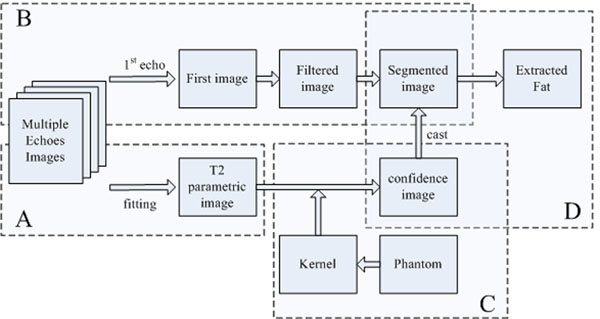
**Procedure of Fat Separation.** The main processing steps include (A) the fitting of parametric image, (B) segmentation of first image, (C) evaluation of phantom and (D) fat extraction and quantification.

As illustrated in Figure.1, serial images derived from multiple spin-echo scans are utilized as input. The processing can be considered in two ways. As diagrammed on the top line, the first image is subjected to operations including smoothing and segmentation. On the bottom line, a weighted least square fitting method is used to produce the T2 parametric image, which is converted into a confidence image based on the Gaussian kernel derived from the phantom study. Finally, the confidence image is cast into the different regions in the segmented image, and groups of fat pixels are distinguished according to their regional confidence scores.

The key techniques used here will be described in detail according to the processing steps including (A) the fitting of parametric image, (B) segmentation of first image, (C) evaluation of phantom and (D) fat extraction.

### A: Fitting of parametric image

To measure the transverse relaxation time for each pixel of the parametric image, a curve is fitted to the decay of intensity with increasing TEs [9]. The non-linear least squares is most commonly adopted [10,11]. For the fitting of fat data, we used the weighted least square method with baseline subtraction and a least point constraint.

#### a1.Fitting model

After testing both mono and bi-exponential models, the transverse relaxation time measurement of fat appears to satisfy a mono-exponential physical model known as: [12]

S_i_(S_0_,T_2_)=S_0_*exp(-Te_i_/T_2_)

with Te_i_=i*te and S_0_ is the pseudo-proton density, which is relative to the true proton density, T1 value and receiver coil response.

#### a2.Weighted least square

To fit a set of experimental data to the parametric model, a cost function and optimization method was selected.

For the cost function, a least square method (LS) is the common way of fitting the curve, which consists in minimizing the quadratic distance Φ^2^ between the fitted curve to the curve represented by the raw data.

Where I_i_ is the scanned intensity of intensity images in i_th_ echo.

Weighted least square method (WLS) takes other factors into account using the weight with merit function:

Where w_i_, the weight of i_th_ point, should represent the confidence of the signal. As the low intensity signals are more likely to be noise and according have less certainty, here w_i_ was simply set as proportion to the intensity (w_i_= I_i_).

Second, for the optimization, a Marquardt-Levenberg algorithm[13], which is specially designed for multi-dimensional non-linear least squares fitting was selected.

### B: Image segmentation of first image

Considerable effort has been devoted to the intesnity image with the aim to segmenting the MRI reconstructed image into tissues. Unfortunately, inhomogeneity, bias, edge blurring and other ill-posed problems require complicated mathematical models. However, unlike the previous methods, only regions within one tissue is our goal for the segmentation (i.e. inhomogeneous fat tissue may be divided into several regions). These regions served as the casting board for the confidence image and fat i ultimately determined by the confidence scores.

To improve the precision our morphological segmentation, bilateral filtering and mean shift methods were used and described below. Here, the image obtained in the first echo is chosen due to its higher SNR relative to the other echoes.

#### b1.MRI filtering

MRI filtering was implemented as a preliminary step to decrease the noise, which replaces the signal of a pixel according to the neighbouring pixels. Filtered image I_f_ can be regarded as a convolution between original image I_0_ and kernel K [14]: I_f_ = K ⊗ I_0_. The filter kernels, typically a matrix of size M*M, represents the number of pixels nearby taken into account. Each element ky (i,j∈[1,M]) at different positions represents the weight at a given point.

Linear and nonlinear are two typical filters used in image processing. In linear convolution filters, the weighting coefficients k_i,j_ only takes into consideration the relative position in kernel K, and remains constant throughout the whole image filtering. Nonlinear filters are relative to the target pixel and the coefficients are calculated as a function of local variations of the signal [15]. In the linear filter class, average and Gaussian filers are often used.

Among the nonlinear filters, the median filter is popular. As well, a selective blurring filter [16] is used [11,17], which emphases the pixels with similar intensity to the target pixel. A bilateral filter [18] is an edge preserving technique proposed recently and has been widely used in image processing. In comparison to the selective blurring filter, not only is the intensity similarity taken into account, but also the spatial similarity after processing with a uniform or Gaussian kernel. A comparison of the effects of different filters on the image data is demonstrated later.

#### b2.Mean shift segments

After smoothing, the first image is segmented into different regions by a mean shift algorithm. Mean shift [19,20] is a nonparametric estimator of density, which can cluster all the pixels according to both their distribution in space and their intensity thus creating a unique feature space. Given a data point x in the first image, after the first shift, the new vector x^(1)^ is:

Where x_i_ are data points around x and the function g() is related to a kernel expression, which defines different influences of x_i_ according to their distance from x. The parameter h is the bandwidth used to control the kernel size.

The shift is iteratively performed until x converges to a stable mode point. Because the similar data points will converge to the same or nearby mode points, all the data points in the first image can be segmented by merging the mode points based on their distances. More details of the mean shift segmentation can be found in [20]

Compared to conventional MR segmentation methods, mean shift is better for analyzing fat images. Mean shift is a local method making it insensitive to non-uniform intensities. Second, unlike the K-mean or EM methods [21], the number of clusters does not need to be predefined. In addition, the bandwidth parameters can be adjusted for the different sizes of kernel functions, which provide a flexible way to define the scale according to research objectives. After this segmentation process is complete, the first image will be divided into regions.

### C:Phantom evaluation

The fitted T2 values of fat pixels in a given region are not exactly the same but rather present a distribution. To evaluate this distribution, a phantom study was done.

#### c1. SNR effect on T2 distribution

From previous literature [22], it is known that the signal to noise ratio (SNR) can influence this distribution, whether or not variances are caused by the animal or acquisition technique. We excluded variance of individual animals, only the factors related to the acquisition technique were taken into account, which included the effect of the imaging protocol and the instrument.

From the aspect of the MRI protocol, SNR is decided by many parameters, including ratio of echo time, spatial resolution, thickness of slices, and receiver band width. From the instrumentation aspect, it is more complicated to understand the physical factors, which include coil setup, magnetic field bias, RF in homogeneities, and phase deviation. Therefore, to evaluate the relationship between SNR and T2 distribution is a complicated task. A practical way is to determine SNR effects is to use a homogeneous sample with the similar acquisition parameters in the same instrument [22].

#### c2. Phantom simulation

**Figure 2:**
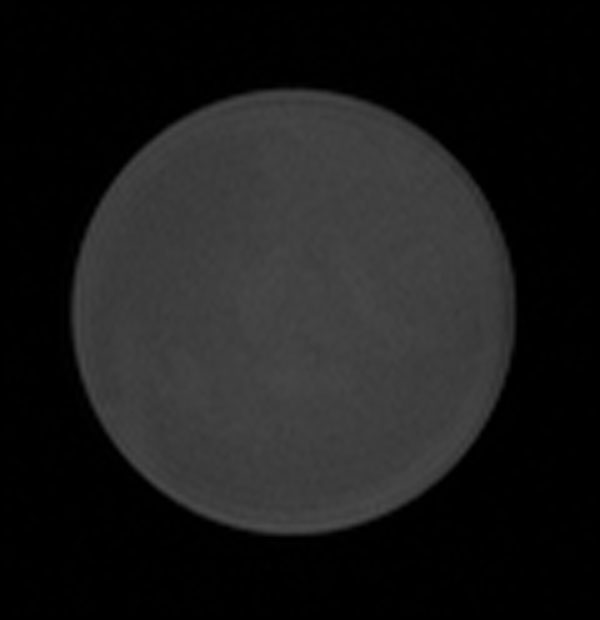
**Lard Phantom** A tube uniformly filled with lard used as the phantom to evaluate the influence of the noise.

A tube filled with uniform lard was used as the phantom (Figure 2) under the identical scan protocol as used in the animals. To simulate the noise effects, white Gaussian noise with increasing normalized variation was added to the original phantom images. With the increasing step size of 0.0001, 100 trails were performed and SNR decreased from 36.48 to 2.46 according to the definition:

SNR=avg(S_1_(S_0_,T_2_))/σ.

Where σ denotes the root mean square (RMS) noise in the background and avg() is the average signal value in lard area.

The noise is assumed to follow a Gaussian distribution when SNR>2 because the actual non-zero average Rician noise will become a quasi-Gaussian distribution in both real and imaginary components [12].

**Figure 3:**
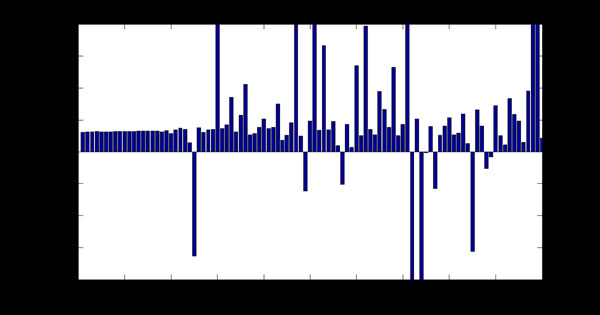
**T2 Test with Phantom** The calculated T2 value in the center area of phantom at the different noise levels.

**Figure 4:**
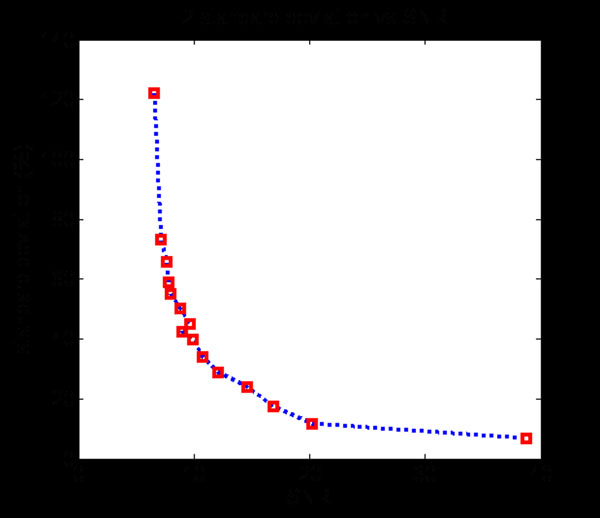
Calibration Curve between SNRs and Standard Deviations.

In the simulated data, weighted least square fitting was utilized for pixels in the center area of tube as defined by a manually selected ROI. The calculated T2 values were recorded as shown in Figure 3 (All values exceeding 200 were trimmed). The results indicated that when SNR<5, the images were corrupted by noise and T2 values were obviously erroneous. Thus, the standard deviations of T2 are only calculated on valid results in Figure 4. An exponential function was fitted and served as the calibration curve for the confidence image.

### D: Fat extraction with confidence image

#### d1. Confidence image

From the phantom evaluation, the measured T2 value in the fat region varies relative to the SNR. Assuming a Gaussian distribution of T2 values, a SNR based Gaussian kernel is utilized here to draw the confidence image. With this kernel, mean and standard deviation need to be determined.

In the T2 parametric image, the T2 histogram shows the different T2 distributions, where different peak mean the different tissues with different biological characters. The T2 value in a peak point means the highest distribution of one region. It is possible to approach the real value. Here, the mode is found by performing a 1-D mean shift method.

With the Gaussian kernel, a weighted filter was used with the calculated T2 value pixel by pixel. For each pixel P, the cast weight w_p_ can be calculated as:

w_p_=exp(-(T_2,p_-T_2,m_)^2^/(2σ_SNR_))

with T_2,p_ is the T2 value of P, T_2,m_ is the mode value P belongs to, and σ_SNR_ means the standard deviation value corresponding to current SNR determined in the phantom study.

**Figure 5:**
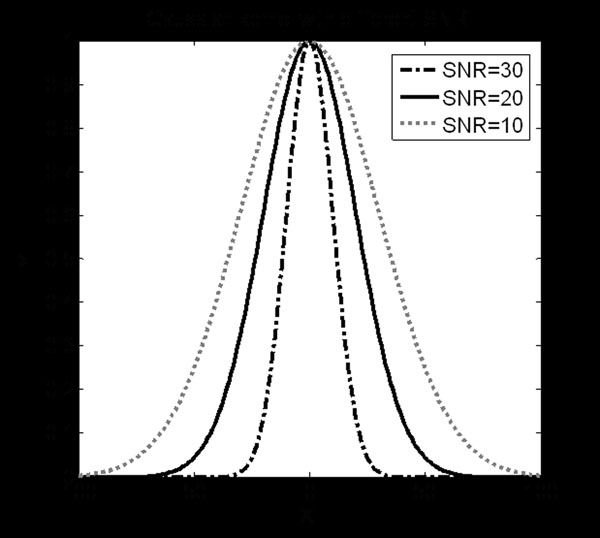
Gaussian Kernel at selected SNRs

From Figure 5, it can be found that the higher SNR, the narrower the kernel will be, which means the T2 value distribution will be closer to the peak value. Also, after the weight, the pixel with the T2 value near the peak value will approach 1, and those with a difference more than four standard deviations will make no sense in the confidence image.

Therefore, the confidence images are defined by the convolution of T2 and SNR based Gaussian kernel: I_c_=I_w_ ⊗ w.

#### d2. Fat extraction

Finally, confidence images are cast into the segmented first images pixel by pixel. And confidence scores of each region are summed by the confidence associated with pixels located in that region.

The probability of each region belonging to adipose tissue is determined by the confidence scores. Fat can be extracted by setting a predefined or real-time threshold on these confidence scores.

## Results

### Imaging protocol

Data were generated on the animal MRI (70/16 Bruker PharmaScan, Germany) with a 16cm diameter bore, field strength 7.05 Tesla, and maximal gradient strength of 400mTesla/m. To acquire intensity images, a Multi-spin multi-echo (MSME) Bruker pulse sequence was used with imaging parameters TR=752ms, NE = 10, TE=8.3ms, FOV=40*40mm, slice thickness=0.9mm.

### T2 fitting

**Figure 6:**
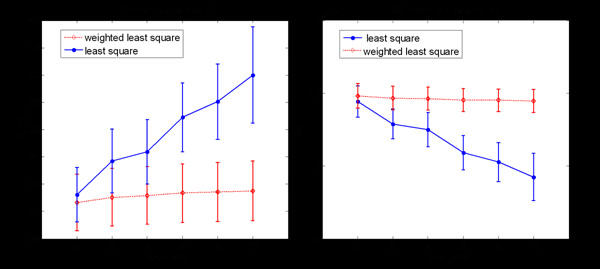
Comparison of Fitting methods

To compare the Weighted Least Square (WLS) to traditional Least Square(LS) algorithms, experiments were performed in the homogeneous phantom with different number of echoes included ranging from 5 to 10. The mean value and standard deviation of T2s and S0 intensities were determined as shown in Figure 6. In this experiment, to increase the reliability results, the baseline subtraction [23,24] method was utilized to remove all the points with intensity lower than a threshold [25]. Also, on the observation that some artefacts results from insufficient sample points, only the pixels with 5 or more valid points after baseline subtraction were calculated.

In Figure 6, it demonstrated that WLS is more insensitive to the number of fitted points than LS method. The WLS method improves the consistency of the calculated T2 values and robustness of final results.

### Filtered image

**Figure 7:**
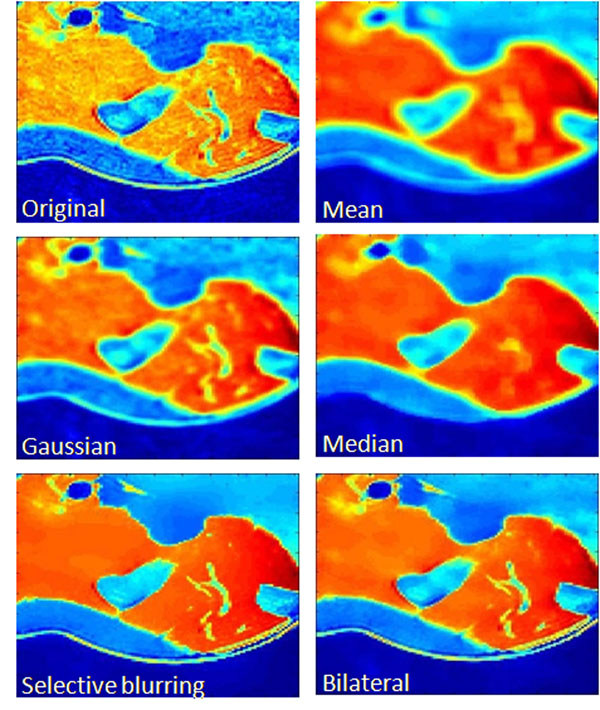
Comparison of Filters

Typical linear and nonlinear image filters are implemented and compared in Figure 7. Here filter matrix size are all 7*7 in a 256*256 image. From the results, we can find the mean and Gaussian filter blurred the edge information; median filter caused some unexpected changes at the sharp edges. The edge preserving selective blurring and bilateral filters appear to be more optimal. For the fat distribution, the fat pixels often appear to be a continuous region, where bilateral filtering is better for taking its spatial info into account.

### Measurement tool

**Figure 8:**
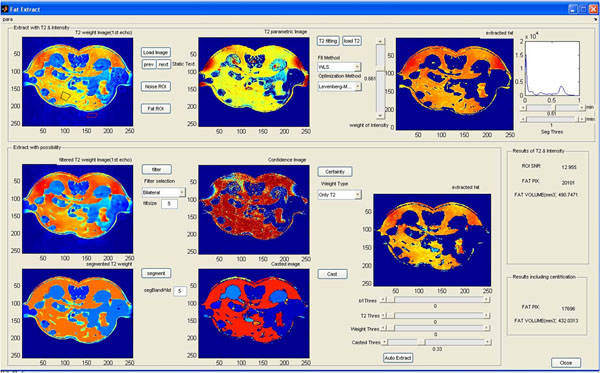
Overview of the Software Tool

We implemented a tool to measure and compare the separated fat region as shown in the screenshot in Figure 8.

In the top part of the graphical user interface (GUI), the traditional method (with only intensity information) is implemented and shown. Either this image or a modified image which is a composite of the original intensity image and weighted parametric image can be displayed.

In the bottom portion of the screen, fat is separated with based confidence images. To provide operator with a clear perspective, all steps are displayed including smoothing (filtering), region segmentation, confidence image and cast image. Operator implement parametric changes can be visualized real-time at each set. We supply the different filters and region segmentation options, and in the confidence image, probabilities are displayed in the pseudo color map (red mean high probability and blue means low probability). Also, based on confidence image, each region in the cast image is colorized in relation to their confidence scores. The effect of varying the confidence threshold on the size of fat region can be observed in real-time. Quantification results are displayed in the right bottom portion of the window.

### Method validation

**Figure 9:**
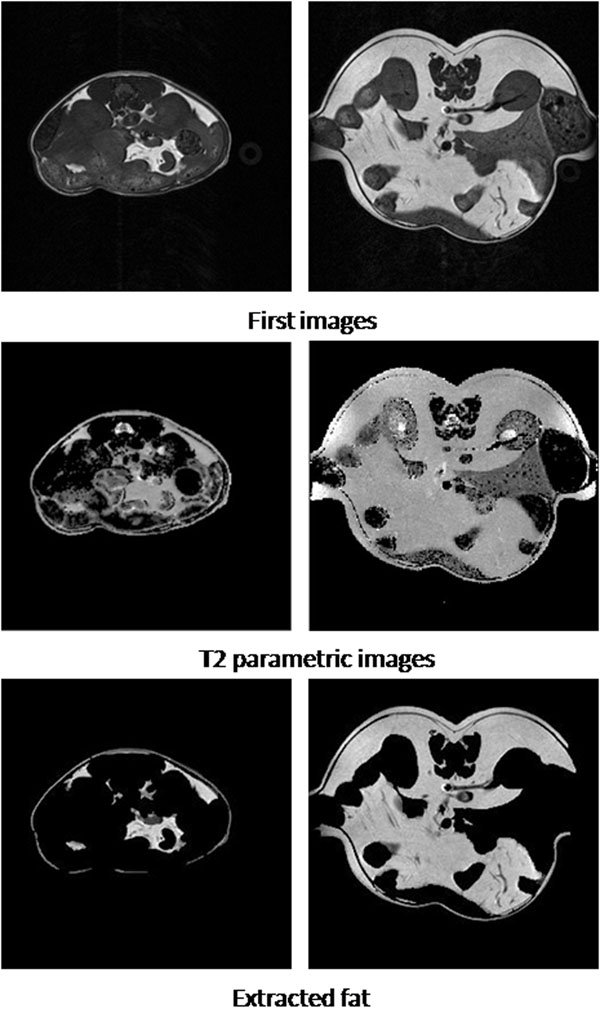
**Fat Extraction** The technique was performed on a lean and a fat mice data separately (SNR nearly 20 to 1). First row displays the first images, and the fitted T2 parametric images are shown in the second row.

To investigate the performance of the proposed method , which combines both first image and T2 parametric images to perform the adipose segmentation (the following is called combination method), we compared the analyses of 100 image slice data acquired from a fat (OB, obese gene) mouse and a thin(WT, wild type) mouse at different resolutions on the 7T MRI. Parts of the results are displayed in Figure 9. In this preliminary research, the manual results from two independent technicians from SAIRC-CHLA(Small Animal Imaging Centre, Childrens Hospital Los Angeles) are considered ground truth.

**Figure 10:**
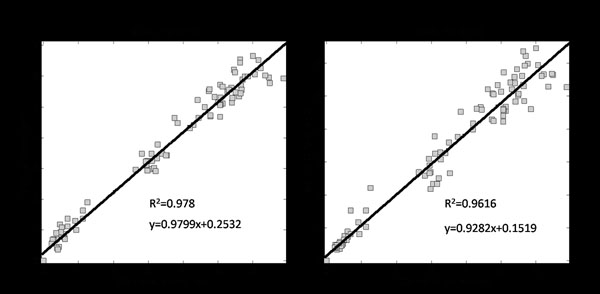
**Correlations of results** The correlation between the results from the combination method and those of two manual operators in a 100 slice study.

There is a high correlation between the results for the manual operations and the proposed method. A linear regression with 95% confidence is displayed in Figure 10.The function is y=0.9799x+0.2532(R^2^=0.978) and y=0.9282x+0.1519 (R^2^=0.9616) respectively. Where the slope of the line function describes the agreement of the measured adipose size, and the r squared indicate how good the linear relationship is. Beyond the total adipose size measurement, a further analysis was performed on the adipose positions in each slice. In order to compare the position, the overlap percentage (OP) of the fat region, which is segmented by combination method (R_1_) and manual operation (R_2_) is calculated as:

The statistical results are performed on two manual references and the average values are listed in Table1.

**Table 1:** Comparison of overlap(%)

mouse type	64*64		128*128		256*256		512*512	
mean	std	mean	std	mean	std	Mean	std

OB	91.37	1.95	93.78	2.09	94.26	2.45	95.41	1.8

WT	66.03	8.8	73	5.9	76.53	4.45	80.78	2.83

From Table1, we find that the performance of WT mouse is obviously lower than OB mouse. This is mainly due to the fact that the WT mouse has relatively less fat, so a small variance in fat will lead to high statistical deviation. Also, the subcutaneous fat in the WT mouse is less obvious, which increase the mismatch of the total fat. The statistical results also reveal that the combination method corresponds more closely to manual method with increasing resolution. In lower resolutions, such as 64*64, the fat tissue is easily mixed with muscle pixels and the partial volume degrades the fat extraction process.

## Conclusions

Combining the intensity and parametric images, we described a method of fat separation with the confidence image and proved it to be feasible with real data. However, limitations still exist and further experiments are needed to define and refine the technique.

The combination method only has the capacity to measure the total fat. Recently researchers [26,27] declared high relationships between the fat distribution and diseases, for example, high levels of visceral adipose tissue have been linked to diabetes. Thus, the different fat types (visceral and subcutaneous) need to be separated for disease related researches. Because subcutaneous fat is always located near the body contour, positional knowledge can be taken into account for further separation. Currently, the method has not been investigated for its ability to discriminate between brown and white fat, which still remains challenging in small animal studies.

In this preliminary research, to test the feasibility of the method, the ground truth for comparison is only based on the in vivo imaging technique. More precise results can be obtained through histopathologic comparison based on the MR Images and sections, which is especially helpful in skinny mouse where adipose is less obvious. To compare the results from different modalities, the co-registration technique is important.

More attention should be paid to the relationship between T2 distribution and different imaging protocols as well as the animal models. Various imaging sequences will be designed by changing the imaging parameters and more animals will be scanned which can be divided into more detailed groups according to their genders and ages.

In conclusion, we report a novel method using both intensity image and T2 parametric image to recognize the adipose tissues on 7T MRI. Proved by experiments on the real data, the method provides a good way to accomplish the adipose separation and may serve as a methodological basis for animal studies.

## Competing interests

The authors declare that they have no competing interests.

## Authors' contributions

Y.T.,S.L.,M.N.,S.R.,R.M. conceived of the project and designed the experiments. Y.T.and R.M. performed the experiments and analyzed the data. Y.T.,S.L., R.M. wrote the paper.
